# Case for diagnosis. Vascular malformations, hemihypertrophy and macrodactyly: Proteus syndrome^⋆^

**DOI:** 10.1016/j.abd.2021.11.012

**Published:** 2023-02-06

**Authors:** Bárbara Elias do Carmo Barbosa, Melissa de Almeida Corrêa Alfredo, Luciana Patrícia Fernandes Abbade, Hélio Amante Miot

**Affiliations:** Department of Infectology, Dermatology, Diagnostic Imaging and Radiotherapy, Faculty of Medicine, Universidade Estadual Paulista, Botucatu, SP, Brazil

Dear Editor,

A one-year-old boy had erythematous-violaceous macules on the left lower limb and trunk since birth (Fig. 1), associated with feet and chest deformities (Figure 2, Figure 3), arteriovenous fistulas, and hypospadia. His personal and family history showed normal delivery at term, with no complications and non-consanguineous parents, with no reports of similar cases in the family, or hereditary diseases.

**Figure 1:**
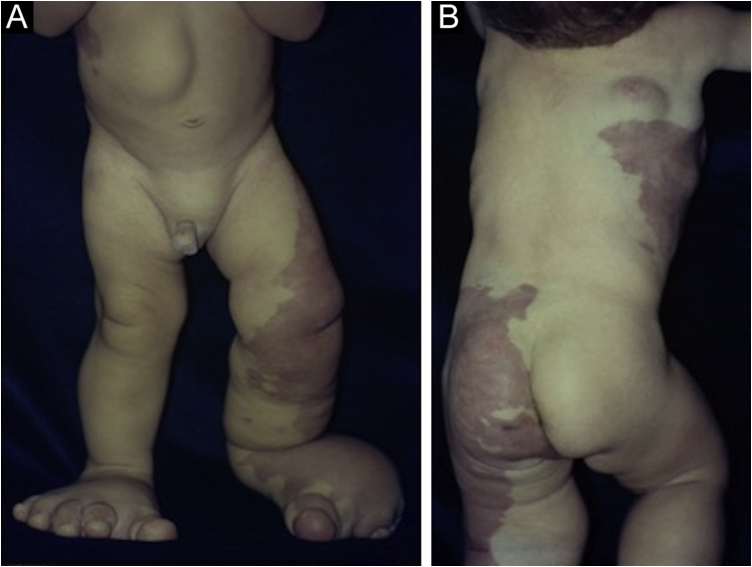
(A) Vascular malformations in the left lower limb. Leg and foot deformities and abdominal tumor. (B) Extensive vascular malformations in the thorax, hip and thighs with mosaic distribution.

**Figure 2:**
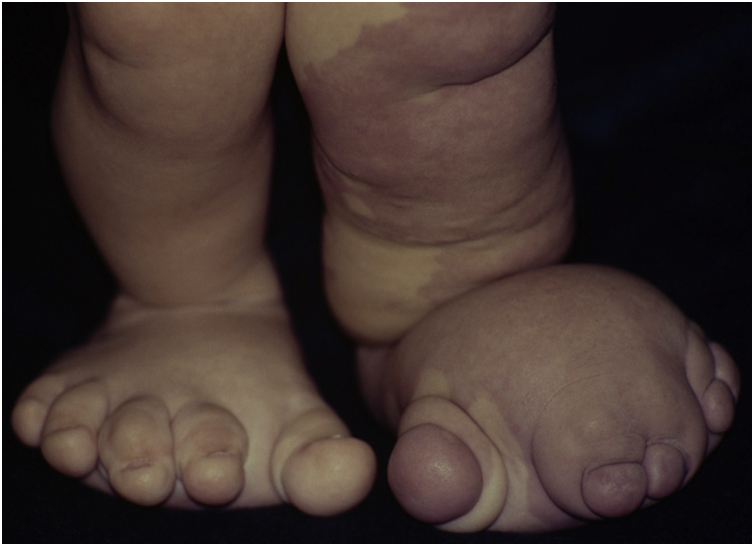
Details of foot deformities with asymmetric gigantism and syndactyly.

**Figure 3:**
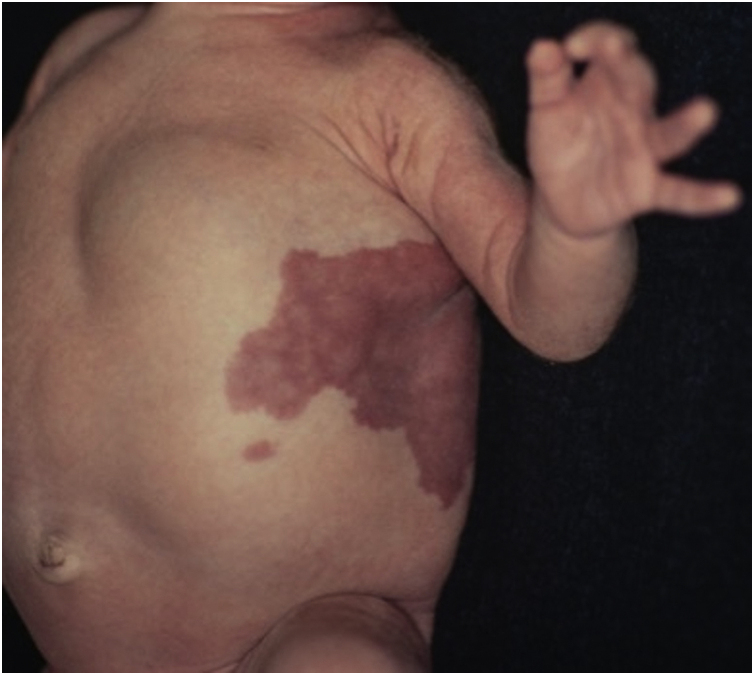
Detail of the vascular malformation in the thorax and thoracoabdominal tumor.

The investigation showed a normocephalic child, cervicothoracic scoliosis, posteriorly rotated ears, straight palpebral fissures, enlarged nasal base, retrognathia, flattened nasal philtrum, high palate, downturned oral commissures, and a palpable mass in the right epigastric region. He also had hemihypertrophy of limbs, enlarged hands and toes (symmetrically), and increased feet volume (left foot larger than the right one) with syndactyly between the second and the third and between the fourth and the fifth toes on the right. Vascular malformations were observed in the left lower limb, dorsum, thorax and genital region, besides linear epidermal nevus on the thorax. He had adequate neuropsychomotor development, without ocular alterations. The genetic analysis disclosed a male karyotype (46, XY), with no qualitative or structural alterations.

## What is your diagnosis?

a Proteus syndrome
b Maffucci Syndrome
c Klippel-Trenaunay-Weber syndrome
d Milroy Disease

## Discussion

Named in 1983 by Wiedmann et al.,^1^ Proteus syndrome is characterized by its polymorphism, variable phenotypic presentations, and mosaic distribution of lesions.^2^ It presents immediately at birth and can affect any organ or system, commonly manifesting with skeletal malformations, overgrowth of connective and muscular tissues, nevi and vascular malformations. Neuropsychological development is usually preserved.^3^

The clinical manifestations are variable, with cases ranging from focal changes (isolated macrodactyly),^4^ to extensive dysmorphism that undergoes changes over time, making the diagnosis and therapeutic approach difficult.^5^ It is considered a rare condition, with an average incidence of 1/10,000,000 births, and less than 150 cases reported worldwide.^6^ It results from a mosaic mutation with somatic activation of the AKT1 oncogene (14q32.3), which is involved in cell-growth signaling pathways,^7^ in addition to being associated with a greater predisposition to neoplasms, deep vein thrombosis, and pulmonary embolism, with a risk of early death.^8^

The diagnosis of Proteus syndrome is based on clinical criteria, making it necessary to differentiate from other hamartoses, such as Klippel-Trenaunay-Weber and Maffucci syndromes (Table 1).^2^

**Table 1:** Main clinical characteristics of Proteus syndrome and its differential diagnoses

**Diagnosis**	**Main clinical characteristics**
Proteus Syndrome	• Asymmetrical growth of one or more limbs, pigmented nevi, cerebriform connective tissue nevi, vascular malformations, subcutaneous tumors, macrocephaly and visceromegaly • Progressive evolution • Facial alterations: dolichocephaly, elongated face, oblique palpebral fissures and/or ptosis, depressed nasal bridge, narrow or wide nostrils, open mouth at rest
Maffucci syndrome	• Cutaneous venous malformations, dyschondroplasia, enchondromas, lymphangiomas, *café au lait* spots
Klippel Trenaunay -Weber Syndrome	• Port-wine stains, vascular malformations, arteriovenous fistulas, hypertrophy of the affected limb (bone and/or tissue)
Milroy Disease	• Lymphedema since birth in lower limbs, varicose veins, limb hypertrophy

Treatment is individualized and multidisciplinary, requiring a psychological and psychomotor evaluation when orthopedic and vascular surgical approaches are indicated. Oral inhibitors of the mTOR pathway (sirolimus 0.1 mg/kg/d) have been reported to be effective in preventing the growth of connective tissue hamartomas.^9^ The use of sirolimus has promising results in controlling symptoms related to limb overgrowth and tumors and should be started early to prevent disease progression.^10^ However, the use of this medication was not available at the time the present patient was evaluated.

This child was followed by the dermatology, pediatrics, psychology, vascular surgery and orthopedics teams for 12 years. At the age of ten, he experienced a worsening of the gigantism of the lower limbs, leading to difficulty in walking and weight loss. The multidisciplinary team decided for a transtibial amputation of the right lower limb and a transfemoral amputation of the left lower limb.

## Financial support

None declared.

## Authors' contributions

Bárbara Elias do Carmo Barbosa: Drafting of the manuscript; effective participation in research orientation; effective participation in propaedeutics; critical review of the literature; critical review of the manuscript; approval of the final version of the manuscript.

Melissa Almeida Corrêa Alfredo: Drafting of the manuscript; effective participation in research orientation; effective participation in propaedeutics; critical review of the literature; critical review of the manuscript; approval of the final version of the manuscript.

Luciana Patrícia Fernandes Abbade: Drafting of the manuscript; effective participation in research orientation; effective participation in propaedeutics; critical review of the literature; critical review of the manuscript; approval of the final version of the manuscript.

Hélio Amante Miot: Drafting of the manuscript; effective participation in research orientation; effective participation in propaedeutics; critical review of the literature; critical review of the manuscript; approval of the final version of the manuscript.

## Conflicts of interest

None declared.

## References

[1] Wiedemann H.R., Burgio G.R., Aldenhoff P., Kunze J., Kaufmann H.J., Schirg E. (1983). The proteus syndrome. Eur J Pediatr.

[2] Capelato Rocha R.C., Estrella M.P.S., do Amaral D.M., Barbosa A.M., Morgado de Abreu M.A.M. (2017). Proteus syndrome. An Bras Dermatol.

[3] Biesecker L. (2006). The challenges of Proteus syndrome: Diagnosis and management. Eur J Hum Genet.

[4] de Almeida H.L., Fiss R.C., Happle R. (2011). Macrodactyly with skin hypertrophy: a minimal form of the Proteus syndrome. An Bras Dermatol.

[5] Pithadia D.J., Roman J.W., Sapp J.C., Biesecker L.G., Darling T.N. (2021). Hypertrichotic patches as a mosaic manifestation of Proteus syndrome. J Am Acad Dermatol.

[6] Sapp J.C., Hu L., Zhao J., Gruber A., Schwartz B., Ferrari D. (2017). Quantifying survival in patients with Proteus syndrome. Genet Med.

[7] Lindhurst M.J., Sapp J.C., Teer J.K., Johnston J.J., Finn E.M., Peters K. (2011). A Mosaic Activating Mutation in AKT1 Associated with the Proteus Syndrome. N Engl J Med.

[8] Zeng X., Wen X., Liang X., Wang L., Xu L. (2020). A case report of proteus syndrome (ps). BMC Med Genet.

[9] Marsh D.J., Trahair T.N., Martin J.L., Chee W.Y., Walker J., Kirk E.P. (2008). Rapamycin treatment for a child with germline PTEN mutation. Nat Clin Pract Oncol.

[10] Weibel L., Theiler M., Gnannt R., Neuhaus K., Han J.S., Huber H. (2021). Reduction of Disease Burden with Early Sirolimus Treatment in a Child with Proteus Syndrome. JAMA Dermatol.

